# Evaluation of cerebrospinal fluid neurofilament light chain levels in multiple sclerosis and non-demyelinating diseases of the central nervous system: clinical and biochemical perspective

**DOI:** 10.17305/bjbms.2021.7326

**Published:** 2022-04-30

**Authors:** Burak Arslan, Gökçe Ayhan Arslan, Aslı Tuncer, Rana Karabudak, Aylin Sepici Dinçel

**Affiliations:** 1 Department of Medical Biochemistry, Faculty of Medicine, Gazi University, Ankara, Türkiye; 2 Department of Medical Biochemistry, Erciş State Hospital, Van, Türkiye; 3 Department of Neurology, Erciş State Hospital, Van, Türkiye; 4 Department of Neurology, Faculty of Medicine, Hacettepe University, Ankara, Türkiye

**Keywords:** Multiple sclerosis, neurofilament light chain, SIMOA, ELISA

## Abstract

The neurofilament light chain (NfL) is a promising biomarker in the diagnosis, prognosis, and treatment response evaluation of neurological diseases. The aims of this study were to compare the cerebrospinal fluid (CSF) NfL levels in multiple sclerosis (MS) and certain non-demyelinating diseases of the central nervous system (NDCNS); to determine the relationship between clinical and radiological features and CSF NfL levels in patients with MS; and to compare the enzyme-linked immunosorbent assay (ELISA) and single molecule array (SIMOA) methods for NfL measurement using paired CSF and serum samples. We retrospectively analyzed the clinical data and performed NfL measurements in CSF and serum samples of newly diagnosed and treatment-naive patients with CNS diseases evaluated between 1 January 2019 and 1 January 2020. Eligible patients were divided into three groups: MS (n = 23), differential diagnosis of MS (n = 19), and NDCNS (n = 42). First, we compared the CSF NfL levels among the three groups using the previously validated CSF ELISA assay. Next, we evaluated the relationship between CSF NfL levels and the clinical and radiological findings in MS group. Finally, we compared CSF and serum samples from patients of the MS groups (paired serum and CSF samples, n = 19) using two different methods (ELISA and SIMOA). The CSF NfL level was the highest in the NDCNS group (1169.64 [535.92−5120.11] pg/mL, *p* = 0.025). There was a strong positive correlation between the number of T2 lesions and CSF NfL level (r = 0.786, *p* < 0.001) in the MS group. There was excellent consistency between ELISA and SIMOA for CSF samples, but not for serum samples. Our results indicated that CSF NfL levels may also be used in the management of NDCNS and that SIMOA is the most reliable method for serum NfL determination.

## INTRODUCTION

In the management of neurological diseases, reliable and easily accessible biomarkers are necessary for establishing the diagnosis, evaluating the prognosis, and monitoring the response to treatment [1]. Ideally, these should be applicable not only to certain, but to various central nervous system (CNS) disease groups, such as inflammatory, neurodegenerative, traumatic, and vascular diseases. In this context, the neurofilament light chain (NfL) is a promising biomarker.

Multiple sclerosis (MS) is a chronic inflammatory, autoimmune, demyelinating disease of the CNS, which has several subtypes, including relapsing-remitting MS (RRMS) and progressive MS (primary progressive MS (PPMS), secondary progressive MS (SPMS)). Even though the presentation of the disease is mostly relapsing-remitting (85%), ~15% of it is progressive from the onset. Both oxidative stress and neuroinflammation play a pivotal role in the pathophysiology of MS. As a result of these pathological events, axonal damage is unavoidable in the end [2,3].

The NfL is a subunit of neurofilaments, which are cylindrical proteins found in the neuronal cytoplasm that maintain axonal stability. Although neurofilaments are present in dendrites and neuronal soma, their expression is particularly high in axons [4]. Because the NfL forms the backbone of neurofilaments and is the most soluble and abundant subunit, it has become possible to reliably measure its levels in biological fluids [5,6]. Under normal physiological conditions, small amounts of NfL are released from axons, which have been reported to increase in older ages [7]. However, NfL release dramatically increases as a result of axonal damage, regardless of the cause. It first passes into the cerebrospinal fluid (CSF) and then into the blood, where its concentration is approximately 40 times lower than that in the CSF [4,8].

As CSF and blood levels of NfL have been shown to increase particularly in neurodegenerative diseases and NfL has been shown to be associated with some disease characteristics, there is an increasing number of studies investigating this relationship. Although CSF is the most valuable specimen type in terms of reflecting physiological or pathological events in the CNS due to its neighborhood with the brain parenchyma, blood samples are more useful in terms of being more easily accessible. Therefore, there is a need for reliable methods to determine blood NfL levels.

Sandwich enzyme-linked immunosorbent assay (ELISA), electrochemiluminescence, and high-sensitive single molecule array (SIMOA) methods have been used in different studies for NfL measurements. While ELISA has limited sensitivity and can reliably measure NfL levels only in the CSF, SIMOA measures serum NfL levels with high sensitivity even in healthy people [9]. Nonetheless, there are still commercially available ELISA kits for serum NfL measurement.

The role of NfL in the diagnosis, prognosis, and treatment response evaluation has been investigated mostly in patients with MS, which is one of the neuroinflammatory diseases [4,10-12], and continues to be investigated with increasing interest not only in MS, but also in spinal cord trauma, Alzheimer’s disease, and frontotemporal dementia [13-16]. However, its role in some non-neuroinflammatory and non-demyelinating diseases of the CNS remains unknown.

Therefore, in this study, we employed clinical and biochemical approaches to investigate the role of NfL both in MS and non-demyelinating diseases of the CNS. The aims of this study were: (1) to compare the CSF NfL levels in MS and non-demyelinating diseases of the CNS, with emphasis on malignant and benign CNS tumors, using a previously validated CSF ELISA kit [17]; (2) to determine the relationship between the clinical and radiological features and CSF NfL levels in patients with MS; and (3) to compare the ELISA and SIMOA methods in terms of reliability for NfL measurement using paired CSF and serum samples.

## MATERIALS AND METHODS

### Study design and patients

This study included a retrospective analysis of the clinical data and post-hoc NfL measurements in CSF and serum samples of newly diagnosed and treatment-naive patients with CNS diseases evaluated between 1 January 2019 and 1 January 2020. Patients who had received steroid treatment in the last month or immunomodulatory therapy in the past three months before sample collection were excluded from the study.

Based on the diagnosis, patients were divided in three groups: newly diagnosed MS, differential diagnosis of MS, and non-neuroinflammatory and non-demyelinating diseases. Since appropriate group selection, proper uniform definitions, and terminology are critical in CSF biomarker studies, the groups were renamed. Definitions in the previously published consensus guideline were used to identify and rename control groups [18]. The differential diagnosis of MS group and the non-neuroinflammatory and non-demyelinating diseases group were renamed as inflammatory disease controls (INDCs) and non-inflammatory disease controls (NINDCs), respectively.

The following criteria were used for the diseases included in the differential diagnosis of MS group (inflammatory disease controls (INDCs)), respectively: (1) Patients who did not comply with the diagnosis of MS clinically and radiologically; and (2) did not meet the McDonald’s diagnostic criteria. In their first clinical attack, these patients prospectively applied to our neurology clinic and were included in this study. First, anti-aquaporin-4 and anti-myelin-oligodendrocyte-glycoprotein antibodies tests were performed in all of these patients whose diagnosis was suspected, and neuromyelitis optica spectrum disorder and myelin oligodendrocyte glycoprotein antibody-associated disease were excluded. Then, diseases that could be confused with MS radiologically were investigated. The main ones were systemic lupus erythematosus, CNS manifestations of primary antiphospholipid syndrome, rheumatoid arthritis, Sjögren’s syndrome, and sarcoidosis. While some of this groups were diagnosed with CNS involvement of certain rheumatological diseases (systemic lupus erythematosus n = 3, rheumatoid arthritis n = 4, Sjögren’s syndrome n = 3, and sarcoidosis n = 2), the rest could not be included in any group and possible clinical isolated syndrome (n = 7) recovered after the first clinical attack and are still being followed up.

### Diagnosis and clinical evaluation

MS diagnosis was determined based on the revised McDonald criteria [2], and disease progression was evaluated using the Expanded Disability Status Scale (EDSS). Relapse was defined as a monophasic clinical episode with patient-reported symptoms and objective findings typical of MS, reflecting a focal or multifocal inflammatory demyelinating event in the CNS, developing acutely or subacutely, with a duration of at least 24 h, with or without recovery, and in the absence of fever or infection [19]. The diagnosis of other non-neuroinflammatory disorders was determined based on clinical and radiological evaluations.

### Data collection

We reviewed the patients’ medical records and collected the following data: demographic (sex, age at sampling, and age at disease onset); clinical (time of disease onset, number of relapses, presenting symptoms, disease subtype for MS, acute and maintenance treatments, and neurological findings at last follow-up); laboratory (serum and CSF examinations); and radiological data (number of T2 lesions). All MRI images were analyzed by an experienced neurologist. The number of T2 lesions was counted on T2-weighted and/or fluid-attenuated inversion-recovery magnetic resonance images. In the brain, T2 lesions >3 mm in diameter were counted in fluid-attenuated inversion-recovery (FLAIR) images using T2-weighted and proton density-weighted spin-echo images as an aid. T2 lesions were also counted in the spinal cord if they were present. Gadolinium enhancing lesions were not counted in all patients, and there are missing data in our data set.

### Sample collection and preparation

Serum and CSF samples were obtained from the Department of Neurology of the Hacettepe University School of Medicine. All lumbar puncture procedures were performed at the same time of the day to exclude the influence of the circadian rhythm. CSF samples with blood contamination were excluded from the study.

Anti-aquaporin-4 and anti-myelin-oligodendrocyte-glycoprotein antibodies were confirmed twice (>1:40 titer) using a commercial fixed cell-based assay (Euroimmun, Lubeck, Germany). This analysis was performed at the time of first diagnosis of MS to exclude certain diseases, such as neuromyelitis optica spectrum disorder and myelin oligodendrocyte glycoprotein antibody-associated disease.

CSF samples were centrifuged at 400 g for 10 min, and blood samples at 2.000 g for 10 min at room temperature within an hour. After centrifugation, samples were stored as aliquots in 1.5-mL polypropylene Eppendorf tubes at −80°C at the Department of Biochemistry of the Gazi University Faculty of Medicine until the time of analysis. Paired CSF and serum samples were transferred to the Neuroimmunology Laboratory of the University Hospital Basel, Switzerland for SIMOA analysis. Other analyzes were performed using ELISA at the Medical Biochemistry Research Laboratory of the Gazi University Faculty of Medicine.

Previously published biobanking procedures were considered as references for processing the CSF samples from all patients and the paired serum samples from certain patients [20]. In Turkey, a body fluid analysis working group has been established within the Turkish Biochemical Society, which is responsible for determining the biobanking procedures for body fluids. The guideline that is going to be published by the Turkish Biochemical Society will also include the biobanking procedures applied in this study (http://www.turkbiyokimyadernegi.org.tr/turkbiyokimyadernegi/vucut-sivilari-analizi-calisma-grubu).

### Measurement of neurofilament light chain levels

For ELISA analysis of CSF samples, we used the NF-light^®^ ELISA Kit (Uman Diagnostics, Umeå, Sweden; catalog number: 10-7002), which has been previously validated for CSF samples. Intra- and inter-assay coefficients of variability (CV%) were <5 and <10, respectively. The measuring range was 100−10.000 pg/mL, with a limit of detection at 33 pg/mL. Before the analyses, all CSF samples were diluted with sample diluent at a ratio of 1:2.

For ELISA analysis of serum samples, we used the Human Neurofilament Light Polypeptide ELISA Kit (Abbexa, Abbexa Ltd, Cambridge, UK; catalog number: abx152468). Intra- and interassay CV% were <10 and <12, respectively. The measuring range was 15.6−1.000 pg/mL, and the limit of detection was < 6.2 pg/mL.

For SIMOA analysis, high-sensitivity SIMOA^®^ NF-Light assay (Quanterix Corp., Billerica, MA, USA) was used for both serum and CSF analysis. All analyses were performed on the HD-X Analyzer™ by running the samples in duplicate. Intra- and interassay CV% were within acceptable limits, with a limit of detection at 0.038 pg/mL. SIMOA analyzes were performed at the Neuroimmunology Laboratory of the University Hospital Basel, Switzerland.

### Ethical statement

This research involving human subjects complied with all relevant national regulations and institutional policies and was conducted in accordance with the tenets of the Helsinki Declaration (as revised in 2013). This study was reviewed and approved by the Ethics Committee of the Gazi University Faculty of Medicine (approval number 33-14.01.2019). Written informed consent was obtained from all participants included in this study.

### Statistical analysis

All analyzes were performed using IBM SPSS Statistics for Windows, version 21.0 (IBM Corp., Armonk, NY, USA). GraphPad Prism software (version 9, San Diego, CA, USA) was used for graphical demonstrations.

Categorical variables were presented as frequencies (n) and percentages (%), while ordinal variables were described by medians and interquartile ranges or means and standard deviations for Gaussian distributed data. The normality of the distribution of numerical variables was examined using the Shapiro-Wilk’s tests. Spearman’s correlation test was used to analyze the correlations of serum and CSF biomarker levels with numeric clinical variables in all patients and within each disease. Mann–Whitney’s test was used to compare two independent groups on a quantitative variable. The Kruskal–Wallis test was used to compare independent k groups (k > 2) on a quantitative variable. The patients’ baseline demographic characteristics were compared using Fisher’s exact test or Wilcoxon’s test. Analysis of covariance (ANCOVA) was performed considering CSF NfL levels as dependent variables, groups (Group 1 = Multiple Sclerosis, Group 2 = INDCs, Group 3 = NINDCs) as fixed variables, and age as a covariate to examine differences between serum biomarker levels among the various groups. Variables with a two-tailed *p* < 0.05 were considered significant.

For comparison of the ELISA and SIMOA methods, the NfL results obtained by the two methods were compared using the paired samples t-test. Consistency was evaluated using Passing-Bablok regression analysis and the relationship between them was expressed as the correlation coefficient. The results were evaluated visually using a Bland–Altman plot.

## RESULTS

The comparison of CSF NfL levels among the three groups and the analysis of their relationship with the clinical and radiological features in patients with MS was performed in a total of 84 CSF samples. There were 23 patients in the newly diagnosed MS group (relapsing/remitting MS n = 19, primary progressive MS n = 1, secondary progressive MS n = 1, clinically isolated syndrome n = 1, and radiologically isolated syndrome n = 1). None of these patients were using immunomodulatory therapy. In the second group, there were 19 patients with a differential diagnosis of MS. The non-neuroinflammatory and non-demyelinating diseases group (n = 42) was divided into five subgroups: malignant brain tumors (n = 4), benign brain tumors (n = 12), hydrocephalus (n = 7), differential diagnosis of headache (n = 9), and other (benign intracranial pressure, etc.) (n = 10). For the comparison of the ELISA and SIMOA methods, a total of 19 paired serum and CSF samples (RRMS = 19) were used.

**FIGURE 1:**
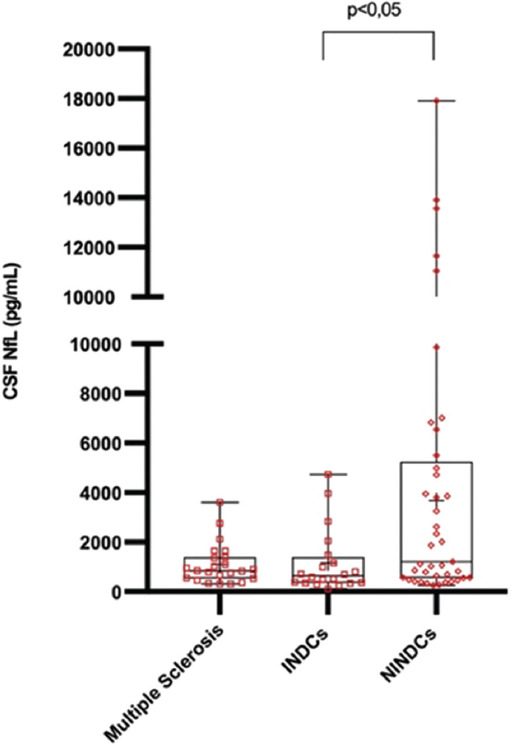
Box-plot graph of the distribution of CSF NfL values in the three groups. Group 1: Multiple sclerosis; Group 2: Inflammatory disease controls (INDCs), Group 3: Non-inflammatory disease controls (NINDCs); CSF: Cerebrospinal fluid, NfL: Neurofilament light chain.

**FIGURE 2:**
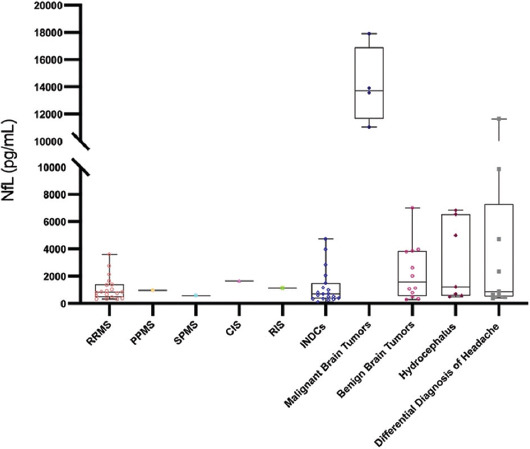
Box-plot graph of the distribution of CSF NfL values in the subgroups. CSF: Cerebrospinal fluid; NfL: Neurofilament light chain; MS: Multiple sclerosis; RRMS: Relapsing–remitting multiple sclerosis; PPMS: Primary progressive multiple sclerosis; SPMS: Secondary progressive multiple sclerosis; RIS: Radiologically isolated syndrome; CIS: Clinically isolated syndrome; Group 2: Inflammatory disease controls (INDCs); Group 3: Non-inflammatory disease controls (NINDCs).

The demographic characteristics of the patients are shown in Table 1. The age at sampling was significantly different among the three groups (*p* = 0.012), and the patients in the non-demyelinating diseases of the CNS group had the highest mean age (45.85±15.62 years). The index immunoglobulin G levels were higher in the MS group than in the differential diagnosis of MS group (1.15±0.70 mg/dL vs. 0.62 ± 0.04; *p* = 0.025). The CSF NfL level was the highest in the non-demyelinating diseases group (1169.64 [535.92−5120.11] pg/mL; *p* = 0.025), with subgroup values as follows: malignant brain tumors, 13099.84 ± 4507.57 pg/mL; benign brain tumors, 3033.50±3028.41 pg/mL; hydrocephalus, 3049.94±2937.34 pg/mL; differential diagnosis of headache, 3502.91 ± 4364.67 pg/mL; and other, 940.60±945.89 pg/mL. The mean CSF NfL level of the malignant brain tumors subgroup was significantly higher than those of the other subgroups (*p* = 0.005). When age was considered as a covariate variable, the main effect of the groups on the CSF NfL value was still found to be statistically significant (*p* = 0.038, F = 3.448, partial eta squared = 0.095). The CSF NfL results and the values of some biochemical parameters are presented in Table 2, Figures 1 and 2.

**TABLE 1:**
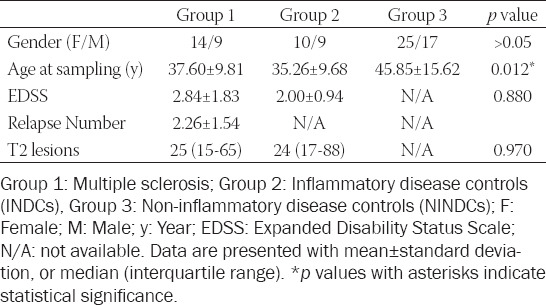
Demographic and clinical characteristics of the patients

**TABLE 2:**
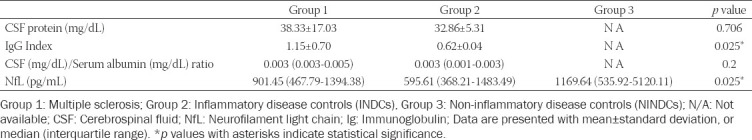
ELISA CSF NfL results and values of biochemical parameters

**FIGURE 3:**
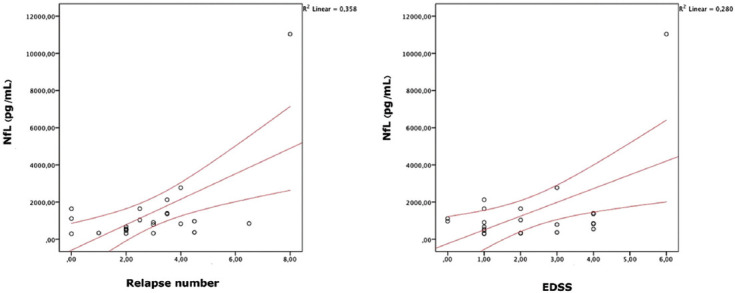
Correlation graphs of CSF NfL values (RRMS patients) with relapse number and EDSS. CSF: Cerebrospinal fluid; NfL: Neurofilament light chain; RRMS: Relapsing-remitting multiple sclerosis; EDSS: Expanded Disability Status Scale.

A statistically significant positive correlation was found between age at sampling and the EDSS score, CSF protein levels, and CSF NfL levels (r = 0.522, *p* < 0.05; r = 0.547, *p* < 0.05; and r = 0.525, *p* < 0.05, respectively; Figure 3). There was also a statistically significant positive correlation between the EDSS score and the number of relapses, CSF/serum albumin levels, and CSF NfL levels (r = 0.673, *p* < 0.05; r = 0.388, *p* < 0.05; and r = 0.598, *p* < 0.05, respectively). There was a strong positive correlation between the number of T2 lesions and CSF NfL levels (r = 0.786, *p* < 0.001). The correlation between biochemical markers and clinical features is shown in Table 3.

**TABLE 3:**
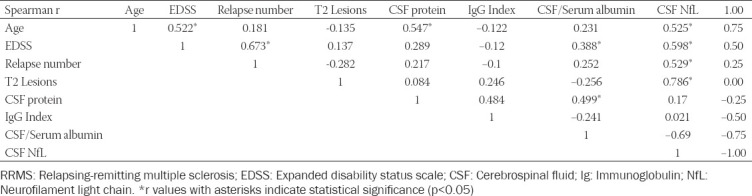
The correlation between biochemical markers and clinical features in RRMS patients

Regression analysis for CSF samples yielded the equation: y = 169.002271 + 0.779003 x (r^2^ = 0.967 (95% CI 0.891−0.990), *p* < 0.001). The intercept A and slope B values were 169.0023 (95% CI 38.1851−469.4667) and 0.7790 (95% CI 0.5870−1.0792), respectively. The paired samples t-test result indicated that there was no statistically significant difference between the two methods for CSF samples (*p* = 0.752). There was consistency between SIMOA and ELISA for CSF NfL according to the slope, intercept, and confidence interval values pertaining to these values. The mean CSF NfL values were 1269.96± 991.10 and 1239.52± 693.05 for SIMOA and ELISA, respectively. For serum samples, based on the regression analysis, the equation was found as y = -126.798808 + 22.433071 x (r^2^ = 0.086 (95% CI, -0.183−0.344), *p* = 0.531). The intercept A and slope B values were -126.7989 (95% CI −693.7511–−26.3872) and 22.4331 (95% CI 12.3235−76.4909), respectively. The paired samples t-test results indicated that there was a statistically significant difference between the two methods for serum samples (*p* < 0.001). There was no consistency between SIMOA and ELISA for serum NfL according to the slope, intercept, and confidence interval values belonging to these values. The mean serum NfL values were 16.83 ± 23.53 and 97.35±71.58 for SIMOA and ELISA, respectively.

## DISCUSSION

In this study, we found that CSF NfL levels were highest among patients with non-demyelinating diseases of the CNS. Furthermore, there was a strong positive correlation between the number of T2 lesions in patients with MS and CSF NfL levels. In addition, our results indicated that SIMOA is the most reliable method for serum NfL determination.

Although many studies have investigated the NfL and its role in disease diagnosis, prognosis, and treatment response [21-23], studies on non-neuroinflammatory CNS diseases are very limited. In the present study, we included patients with non-demyelinating diseases of the CNS, such as malignant and benign brain tumors, which have been less studies. We found that CSF NfL levels were significantly increased in patients with malignant tumors compared to those in other CNS diseases. Although this could be expected, our results are valuable in that this analysis has not been performed before. This finding indicates that patients with brain tumors may be followed up in a minimally invasive manner by monitoring the NfL levels using high-sensitive SIMOA.

Furthermore, we found that CSF NfL levels in patients evaluated for differential diagnosis of headache were lower in patients who were pathologically diagnosed with a benign lesion. This suggests that CSF NfL levels may be used to differentiate benign from malignant lesions. In addition, CSF and serum NfL level monitoring may be helpful to indicate the existence of a pathological brain lesion in patients with clinical but no radiological findings. This may enable early suspicion and diagnosis of malignant tumors that may be overlooked radiologically based on clinical findings and NfL levels.

Our results also showed that CSF NfL levels were correlated with age, EDSS scores, the number of relapses, and the number of T2 lesions in patients with MS, which is in agreement with the findings of previous studies [24-26]. This confirmed the accuracy of our study design.

At present, there are many commercially available ELISA kits from different companies that measure serum NfL levels. In this study, we compared the ELISA and SIMOA methods and found that there was very good consistency between them in terms of CSF NfL levels. However, there was no consistency between the two methods for serum samples. This indicated that commercially available ELISA kits for serum NfL measurement are not reliable and that SIMOA remains the most reliable and precise method for serum NfL measurement. SIMOA’s superiority has been shown in previous studies [9].

It has been shown in the previous studies that NfL is released in increased amounts with aging [27,28]. Especially in studies with NfL, age should be taken as a covariate or adjusted for prior to statistical analysis. This study compared the CSF NfL levels between groups by first excluding age as a covariate variable and then using ANCOVA to examine the difference when age was included in the model. There was still a significant difference when age was taken as a covariate. However, this may also be due to the groups’ relatively low number of samples. In addition, contrary to the previous studies, Van den Bosch et al. did not observe any effect of age on CSF NfL levels in MS [29].

Pathologically, MS is characterized by different types of lesions that can be staged by the presence and morphology of microglia/macrophages in relation to demyelination. Previous studies investigated the role of CSF and serum/plasma NfL measurements in MS by combining the conventional MRI and clinical features. What is new about axonal damage is the question if neuropathological properties of inflammatory lesion activity, neuroaxonal injury, or neurodegeneration correlate with axonal damage, as reflected by CSF NfL levels in MS [29,30]. Van den Bosch et al. stated that CSF NFL is a biomarker that reflects inflammatory white matter lesion activity and is associated with disease progression in MS using postmortem brain tissue and CSF NfL. Besides, contrary to previous studies, they did not observe an effect of age on CSF NfL levels in MS [29]. When age was added as a covariate factor in our study, no effect on CSF NfL levels was observed.

The importance of neurobiomarkers was highlighted in a recent article [31]. In this context, the Turkish Biochemical Society has been preparing a guideline that will be a source for clinicians and researchers for neurobiomarker research. The present study is the first study conducted in Turkey using the SIMOA method and the NfL ELISA kit previously validated in CSF samples of different patient groups.

Our study had some limitations. First, we could analyze only a proportion of the CSF samples using SIMOA due to the related financial burden. However, we plan to expand this study in a larger patient cohort by introducing this ultrasensitive method, which has a great role in neurobiomarker research, in our country. Second, due to ethical considerations in our country, we could not obtain CSF samples from healthy controls for comparison to those of different patient groups. Third, in patients with brain tumors, only CSF samples obtained before surgery were available. Therefore, we could not compare the pre- and post-operative CSF NfL levels. Besides, active Gadolinium enhancing lesions represent a more robust radiological activity parameter. We could not include these parameters because of missing data.

## CONCLUSION

This was the first study in Turkey to examine NfL levels in CSF and serum samples using the SIMOA method. Our results indicated that CSF NfL levels may be used not only in the follow-up of demyelinating, but also in the management of non-demyelinating CNS diseases. Moreover, we have once again shown that SIMOA is the most reliable method for serum NfL levels determination. Nonetheless, further studies with larger samples are needed to investigate NfL levels in the serum and CSF and their role in non-demyelinating CNS diseases, such as metastatic or non-metastatic brain lesions.
