# Corrigendum: Evaluation of cerebrospinal fluid neurofilament light chain levels in multiple sclerosis and non-demyelinating diseases of the central nervous system: clinical and biochemical perspective

**DOI:** 10.17305/bjbms.2022.8354

**Published:** 2023-03-16



Corrigendum to: Evaluation of cerebrospinal fluid neurofilament light chain levels in multiple sclerosis and non-demyelinating diseases of the central nervous system: clinical and biochemical perspective [1].

This corrigendum corrects funding information to: This project was partly supported by Gazi University Scientific Research Projects Coordination Unit (BAP) (project code: 01/2019-38). The authors would like to thank Gazi University for funding this project. The authors sincerely apologize for the error and confirm that this correction does not change the conclusion of the article.
